# Role of the biological active components of human milk on long-term growth and neurodevelopmental outcome

**DOI:** 10.1186/s13052-024-01773-z

**Published:** 2024-09-30

**Authors:** Chiara Peila, Lorenzo Riboldi, Alessandra Coscia

**Affiliations:** Neonatal Unit, Department of Public Health and Pediatrics, University of Turin, Turin, Italy

**Keywords:** Human milk, Biological active components, Human milk biomarker; neurodevelopmental outcome

## Abstract

Human Milk is the best option for infant feeding; and for this reason, it should be promoted, protected, and supported. HM is an individual-specific-dynamic biofluid, characterized by an extreme variability in its composition. A wealth of literature has investigated how HM is related to healthy development. An association between HM composition, including nutrients and growth-related hormones as well as other bioactive components, and short-term and long-term infant outcomes could support this statement; however, the evidence is limited. In fact, HM composition is difficult to examine as it is dynamic and changes within a single feed, diurnally, according to stage of lactation and between and within populations. The aim of this review is summarizing only the innovative knowledge on the association between HM composition and long-term outcomes: infant growth and neurodevelopment. In this specific contest, macronutrients and historical biological component with well recognized effect were excluded (i.e. LCPUFA, DHA, iodine). Revised articles have been found in MEDLINE using breast milk-related outcomes, neurodevelopment, infant growth, breast milk-related biological factors, biomarkers, biological active components, and constituents as keywords. Moreover, we focus our search on the latest research results.

## Introduction

Human milk (HM) is a peculiar food owing unique properties and resulting the ideal nourishment during neonatal period for the growing infant [1–4]. HM is an individual-specific-dynamic biofluid, characterized by an extreme variability in its composition, with regard to both nutritional and bioactive components [5]. Influences on compositional differences include time of lactation, length of gestation, maternal diseases, genotype and diet [6]. From an evolutionary perspective, its composition has evolved over time to provide the infant a well-balanced nutrition and protection against potential infectious pathogens while the neonatal immune system completes its development. A wealth of literature has investigated how HM is related to healthy development. HM is recommended as the optimal nutrition not only for the full-term infants but also for the preterm infants, particularly those born extremely preterm [1]. HM consumption has been reported to significantly reduce **complications associated with prematurity**, including necrotizing enterocolitis, retinopathy of prematurity, bronchopulmonary dysplasia and late-onset sepsis. Furthermore, HM feeding promotes feeding tolerance with a reduced number of days needed to achieve the full enteral feeding [1]. **Long term benefits** are characterized by low risk of re-hospitalization due to respiratory diseases, and promotion of neurodevelopment and neurocognitive outcome. Several data highlight that breastfeeding is related to **positive cognitive outcome**. Specifically, the intelligence quotient in ever breastfed/longer breastfed infants has been reported to be 3.4 (95% CI 2.3–4.6) points higher than that of never breastfed/shorter breastfed infants. Of note, the 16 studies included in the meta-analysis controlled for several potential confounding factors; furthermore, even considering the adjusted data for maternal intelligence quotient, breastfeeding remained associated with an increase in 2.6 points in the intelligent quotient [7-9]. Recent research has also indicated a potential cumulative effect of human milk feeding on promotion of fat free mass deposition, which, in turns, appears to promote cognitive outcome. In terms of **growth**, breastfeeding is associated with lower rates of infancy weight and length gain after the age of 2 months, compared with formula feeding, and there is suggestive evidence that breastfeeding is protective against later overweight and obesity [10–11].

An association between human milk composition, including nutrients and growth-related hormones as well as other bioactive components, and short-term and long-term infant outcomes could support this statement; however, the evidence is limited. In fact, HM composition is difficult to examine as it is dynamic and changes within a single feed, diurnally, according to stage of lactation and between and within populations. However, in this multiplicity of biologically active components that are being discovered, it is very important for the clinician to know their effects, especially in the long term. This can allow important nutritional adjustments to be made, especially in fragile populations such as the preterm newborn.

The aim of this review is summarizing **only the innovative knowledge on the association between HM composition and long-term outcomes: infant growth and neurodevelopment.**

## Materials and methods

This is a narrative review. The literature review was per- formed by conducting electronic searches in PUBMED (National Library of Medicine (United States), National Center for Biotechnology Information, available from https://pubmed.ncbi.nlm.nih.gov) and EMBASE (Elsevier Ltd., available from https://www.embase.com). The search strategy involved a 4-step process using ‘‘MeSH’’ and ‘‘Title/Abstract’’ terms. The first 3 searches were performed separately, and these were followed by the fourth search, which combined the results from the first 3 searches in each database to obtain the articles that were to be screened for relevance and subsequent review. The first search was on the following words: human milk OR breast milk OR breastmilk OR breast fed OR breastfed OR breastfeed; the second was on biological active components OR composition OR micronutrients OR biomarkers; the third was auxological outcome OR neurodevelopmental outcome OR infant growth. This procedure was followed for all the databases, except for variation in the search terms specific to a single database. The investigators determined eligible studies by screening the titles and available abstracts of all of the studies compiled from the final electronic database search. Bibliographies from studies included in the systematic review were also examined for additional applicable studies.

To minimize the bias of this systematic review, the authors (C.P., L.R., and A.C.) evaluated the list of entries separately, according to predetermined inclusion/exclusion parameters, and finally consensus was reached by these authors. The inclusion criteria were primary (original) research published in a peer-reviewed journal; studies including only HM (either preterm or term HM and colostrum, transitional, or mature HM). The exclusion criteria were the study design was a review, letter to the editor, or conference paper; the article included animal milk. In this specific contest, macronutrients and historical biological component with well recognized effect were excluded (i.e. LCPUFA, DHA, iodine, zinc). Moreover, we focus our search on the latest research results (Studies published between 2010 and 2023).

## Results

### Auxological outcomes

The main results on this topic are summarized in Table 1.

**Table 1:** Summary of human milk biomarker and auxological outcomes

Biological Components	References	Possible Effect
**Human milk oligosaccharides**	13,14	No significant differences in infant anthropometric measurements
**Adiponectin**	16	HM adiponectin concentrations might be associated with greater weight gain and higher fat mass in the offspring
	17	HM adiponectin concentrations were predictors in the model of infant fat mass gain.
**Insulin**	17	HM insulin negatively associated with WLZ trajectory among infants of NW mothers. HM insulin concentrations were all predictors in the model of infant fat mass gain.
	18	No associations between human milk insulin concentrations and infant growth or body composition.
**Cortisol**	19	High cortisol concentration had lower BMI at 2 years
**Insulin-Like Growth Factor 1**	21	Early expressed breast milk does not affect IGF-1 plasma levels in infants with gestational age < 31 weeks.
	22;23	No significant differences were observed in concentrations of IGF-1 between two groups (overweight/obese vs. normal weight infants).
	24	IGF-1 was higher in a group of infants with high weight gain, compared with infants with low or normal weight gain.
	25	Higher milk IGF-1 was associated with higher weight at 13 months (*p* = 0.004) but lower weight at 3 (*p* = 0.011) and 5 years of age (*p* = 0.049).
**Transforming growth factor beta-1**	26	TGF-β1 was significantly higher in infants 3–6 months with a significant difference regarding infants’ weight and length
**Interleukin-6**	18	IL-6 were associated with lower infant weight, relative weight, and lean mass at first month of age
	27	no associations in the role of maternal BMI, sex and stage of lactation on HM IL-6 and their associations with infant body composition.
**Tumor necrosis factor-alfa**	18	Negatively associated with infant total lean mass at first month of age
	27	no associations in the role of maternal BMI, sex and stage of lactation on HM IL-6 and their associations with infant body composition

#### Saccharide

Human milk oligosaccharides (HMOs) are bioactive components unique for human milk. HMOs consist of a lactose molecule, which can be elongated with other disaccharides through different enzymatic linkages [12]. Two studies investigated the association between HMOs and infant growth, and found no significant differences in infant anthropometric measurements [13, 14]. 

### Protein

#### Hormones

Hormones in HM are likely to have significant influence on infant growth and body composition. The most researched HM hormones are *adiponectin and leptin*, but currently a negative association between both hormones and infant growth exists. The evidence was stronger for adiponectin, whereas results on leptin were less consistent [15]. 

Brunner et al., found human milk adiponectin concentration (6 weeks postpartum) to be negatively associated with infant growth up to 4 months of age. To the contrary, the authors found a positive association between human milk adiponectin concentration (4 months postpartum) and infant weight gain (4 months to 2 years of age) and adiposity at 2 years of age [16]. Recent research evaluated the effects of HM leptin, adiponectin, ghrelin, insulin, and some cytokines on infants’ growth and body composition measured from birth to 4 months of life. The authors detected an influence played on growth trajectory and/or infant fat mass gain. Specifically, HM insulin was associated with a downward weight-for-length Z-score (WLZ) trajectory with a variability within the normal range of growth. HM glucose and ghrelin concentrations were both associated with slower rates of fat-free mass (FFM) deposition and also mean HM adiponectin concentrations were inversely correlated with fat mass gain. On the other hand HM with a higher n-6:n-3 ratio was predictive of faster fat mass gain [17]. In an exploratory study, Fields et al. found no associations between human milk insulin concentrations and infant growth or body composition [18]. 

Among many of the bioactive factors, breast milk contains glucocorticoids (GCs): cortisol, cortisone, and corticosterone GCs are transferred to BM through simple diffusion. They help to regulate the infant’s cortisol levels and various physiological processes and promote healthy growth and development.

Cortisol concentration in HM has been less studied. However, HM cortisol could potentially influence infant growth as it is involved in storage and metabolism of glucose. In a small cohort (n:51), HM cortisol concentration was measured in a single HM sample at 3 months postpartum [19]. Infants receiving HM with high cortisol concentration had lower BMI at 2 years of age and the associations were more pronounced in girls compared with boys. It was suggested that exposure to cortisol in HM enhancesenhance programming of metabolic function and stimulates maturation of the gut. Finken et al. were however reluctant to accept these results and argued that as cortisol concentrations in HM have a strong diurnal pattern, it is necessary to have frequent milk sampling over 24 h in order to get a reasonable measure of infant cortisol exposure [20].

#### Growth factors

**Insulin-like growth factor-1 (IGF-1)** plays a key role in regulation of growth during the first years of life. IGF-1 is present in human milk; however, the degree of gastrointestinal absorption in the infant is still discussed [18]. Connection between breastfeeding and IGF-I concentration in infants, both in the digestive tract and blood serum is demonstrated. Furthermore, there is limited evidence of an association between HM IGF-1 and infant growth. In a study by Serrao et al. plasma IGF-1 from preterm infants who received mostly HM, which is known to have high concentrations of IGF-1, was compared with a preterm infant group who received mainly pasteurized milk, where IGF-1 is inactive, and some infant formula, where IGF-1 is not detected. The authors reported no difference in infant plasma IGF-1 between the two groups. As discussed by the authors, possible explanations for this could be (i) human milk IGF-1 concentrations were too low to influence serum levels, (ii) no intact absorption of IGF-1 to the portal circulation, or (iii) IGF-1 was metabolized in the liver [21]. Others 2 studies compared the mothers’ human milk hormone concentrations, including IGF-1, in two groups of breastfed infants: 40 overweight/obese and 40 normal weight infants. No difference in HM IGF-1 between the two groups was reported [22, 23]. However, a previous study found that human milk IGF-1 was higher in a group of infants with high weight gain, compared with infants with low or normal weight gain [24]. This higher concentration in the cases of breastfeeding can be linked with the improved regulation of growth stimulation and guide differentiation in the first years of life. These regulations may define infant growth in terms of fat accumulation, and therefore BMI in the first years as suggested by Gila Diaz et al. [25].

**Transforming growth factor beta-1 (TGF-β1)** may play a role in infants’ growth and development. In a study by Alsharnoubi et al., TGF-β1 was significantly higher in infants 3–6 months than those < 3 months (*p* = 0.010); also there was a significant difference regarding infants’ weight and length with average weight and length (*p* = 0.042) and (*p* = 0.009), respectively [26].

#### Cytokines

Bioactive components, such as cytokines, are also present in human milk and have been hypothesized to play a role in infant growth. A small study by Fields et al. found that human milk interleukin-6 and tumor necrosis factor (TNF)-a were negatively associated with infant total lean mass at first month of age, and interleukin-6 was further negatively associated with infant weight and fat mass change in the first month of life [18]. A subsequent study reported that no associations or interactions were observed in the the role of maternal body mass index (BMI), sex and stage of lactation (month 1 vs. 6) on HM IL-6 and TNF-α and their associations with infant body composition [27].

### Neurodevelopmental outcomes

The main results on this topic are summarized in Table 2.

**Table 2:** Summary of human milk biomarker and neurodevelopmental outcomes

Biological Components	References	Possible Effect
Brain-derived neurotrophic factor	28	Higher levels of serum BDNF in breastfed infants, compared to formula fed infants
	29	concentrations levels were higher (*p* < 0.05) in the preeclampsia group as compared to controls
	30	levels were lower at 1.5 months (10.5%) in the preeclampsia group as compared to control group
	31	Serum and milk BDNF levels are higher in epileptic infants than in controls and may be used as a marker of disease severity.
	32	present in all samples of human milk
Glial cell line-derived neurotrophic factor	32 33	present in all samples of human milk detected in HM even up to 3 months of lactation
S100 B	32	present in all samples of human milk
	36	present in preterm milk as well as in term milk during maturation degree
Activin A	39	Detectable in all the measured milk samples, either in term or preterm milk samples
	40	No significant differences between preeclamptic and normotensive women.
Milk Fat Globule Membrane (supplementation to diet)	46	accelerated neurodevelopmental profile at day 365 and improved language subcategories at day 545.
	47	Higher hand and eye coordination IQ, performance IQ, and general IQ.
	48	no differences in growth and neurodevelopment were found between groups.
	49	Higher cognitive score

### Protein

#### Neurotrophic factors

Brain-derived neurotrophic factor (BDNF) glial cell line-derived neurotrophic factor (GDNF), and S100 B protein, significantly impacts the nervous system’s final formation. BDNF is a small dimeric protein belonging to the neurotrophin family, which is widely expressed in the brain. BDNF is a growth factor that plays an important role in neurodevelopment and it is conflicting associated with dietary DHA. Nassar et al. reported higher levels of serum BDNF in breastfed infants, compared to formula fed infants, though breast milk was not measured as a potential source of the BDNF [28]. Limited studies have assessed BDNF in human milk, with a 100-fold difference in concentrations reported between studies but BDNF was always detected [29–31]. Glial cell line-derived neurotrophic factor (GDNF) is a distant member of the transforming growth factor β superfamily that was originally isolated from the rat B49 glial cell line. This protein is a glycosylated, disulfide-bonded homodimer with a molecular weight of 33–45 kDa [32]. BDNF and GDNF with another one from this family, ciliary neurotrophic factor (CNTF), are present in the detected concentration in HM even up to 3 months of lactation [30, 32, 33]. The S100B is calcium-binding protein, characterized by a low molecular weight and a special EF-hand structure that is homodimeric wherein each beta monomer is approximately 10.5 kDa. S100B levels detected in milk were considerably higher when compared to those of other biological fluids. This finding is consistent with the notion that calcium binding proteins are highly concentrated in a biological fluid such as milk in which calcium is abundant [34, 35]. S100B, BDNF, and GDNF have an important role in the development and maintenance of the nervous system, and in neuronal survival and proliferation. What is worth highlighting, the levels of S100 B protein and GDNF elevate with the lactation time [36]. Moreover, these proteins have been implicated in the modulation of learning and memory [28–33, 35]. 

Activin A is a dimeric protein belonging to the transforming growth factor beta (TGF-beta) superfamily and its receptors are widely distributed in the brain. In particular, Activin A has been shown to: (i) exert a neurotrophic role being involved in growth and differentiations of many CNS target cell-types; (ii) play, in vitro and in vivo, a beneficial role in recovery and survival of neurogenic cell lines and retinal neurons decreasing ischaemic brain injury; (iii) exert CNS protection from antidepressant treatment side-effects. Activin A has been also detected in HM supporting its role as a growth factor [35, 37–40].

#### Milk fat globules

Milk Fat Globule Membrane (MFGM) originate in the cytoplasm of the mammary gland epithelial cells, where triacylglycerols (TAGs) are packed into droplets surrounded by a phospholipid monolayer derived from the endoplasmic reticulum. These lipid droplets reach the surface of epithelial cells and then are extruded after fusion with the apical plasma membrane, which confers a peripheral bilayer. Therefore MFGs consists of a triple layer of phospholipids/cholesterol with bioactive lipids and proteins located in different layers of the membrane. The specific feature of the MFGM is the interaction between cholesterol and highly saturated sphingolipids, which makes the structure rigid and allows the bioactive proteins to resist pepsin digestion and to intact reach the small intestine [41–43]. MFG is a complex structure made from several components:

*Lipids.* Core lipids consist mainly of TAGs, which represent almost all of the milk fats and provide more than half of the infant’s energy requirement.

*Proteins.* MFGM proteins account for about 1-4% of total milk proteins. About 200 proteins have been identified and characterized. The most represented (major proteins) are classified into non-glycosylated. (e.g. adipophilin (ADPH) and fatty acid binding proteins (FABS) and glycosylated (e.g.butyrophilin, mucins, xanthine oxidoreductase ). In addition to these proteins, a large number of proteins present in smaller quantities (minor proteins) have been identified, among which the best characterized are α-lactalbumin, β-casein, lysozyme, lactoferrin, osteopontin, Immunoglobulins (e.g., IgA α-chain) [41–44]. Numerous studies have shown that MFG, in addition to purely nutritional functions (supply of very energetic nutrients), have a series of biological functions, which probably depend on the structure as a whole and on the synergic interaction between the different components [43].

The biological functions include development of the neonatal gut and developing intestinal microbiota, reduce the risk of infection in the newborn and promote immunological maturation of the intestine.

Particularly intriguing are the studies concerning the role of MFGMs in brain development [43, 44]. There is an increasing body of evidence from animal models that emphasizes the role of MFGM in providing many components that can support neurodevelopment, such as sialic acid (from gangliosides), sphingomyelin and choline, by improving dendritic pruning and diffusion. Furthermore, polysialic acid is an element of neural cell adhesion molecule (NCAM) which plays a key role at synaptic level. In humans, low maternal levels of choline during pregnancy are demonstrated to be related to poor cognitive development of the offspring [45]. 

Preclinical studies performed in animal models (piglets, rats) have demonstrated that provision of polar lipids and sialic acid improves neonatal brain development (in particular hippocampus and corpus callosum) which is related to better performances in functional tests (learning ability and memory). In addition, clinical trials have been published on neurodevelopmental outcomes after supplementation of MFGM (with or without other bioactive components) in infant formula.The more recent trials show that, at 18 months of age, infants fed with the supplemented formula had higher test scores in multiple domains (cognitive, language and motor) of the Bayley Scales of Infant Development [46, 47] or significantly improved visual function [48, 49]. However it is very difficult to establish whether these effects are attributable to MFGMs alone (and to which component) or whether they are attributable to other bioactive components added to the formula [39−40].

## Discussion and conclusion

Breast-feeding is considered to be the best option for infant feeding; and for this reason, it should be promoted, protected, and supported as the only nutritional source for infants [1]. According to several observational studies, early nutrition can influence both short- and long-term newborn’s health outcomes. Especially in preterm neonates admitted to neonatal intensive care unit, HM could sustain neonatal development, reducing long-term growth failure and neurodevelopmental disease [1–4]. 

Some evidence, investigating the effects of HM in VLBW’s outcome, reported that neonates fed with HM gain less weight during their hospitalization in NICU but this relative undernutrition do not negatively affect their neurodevelopment even considering long-term effects [50–57]. Several reasons could contribute to determine this outcome, especially on premature subjects with immature brain destined to a rapid cerebral development; in this period, this tissue results highly vulnerable to oxidative stress, inflammation, and nutrient deficiency.

In this setting, HM provides bioactive molecules, anti-inflammatory components, antioxidants, growth factor, human milk oligosaccharides. However, limited evidence is available on how human milk composition influences infant growth and body composition. The few studies available suggest functional implications of human milk composition and significant importance for infant growth and neurodevelopmental outcomes. A possible explanation is that HM composition is difficult to examine as it is dynamic and changes within a single feed, diurnally, according to stage of lactation and between and within populations.

Regarding the auxological outcomes several studies underlined how Bioactive components in HM may contribute to regulation of partitioning of body composition. For example, human milk oligosaccharides (HMOs) have no significant differences in infant anthropometric measurements. As per the Hormones in HM, they are likely to have significant influence on infant growth and body composition even though their associations with infant growth and adiposity are sparse.

HM protein, adiponectin and insulin concentrations, and n-6:n-3 ratio were all significant predictors in the model of infant fat mass gain.s. Higher adiponectin levels in breast milk might be associated with greater weight gain and higher fat mass in the offspring up to 2 years.On the other side, HM insulin negatively associated with WLZ trajectories and Greater milk leptin, IL-6 and TNF-α were associated with lower BMIZ (BMI-for-age z-score), relative weight, and lean mass.

Regarding the neurodevelopmental outcomes studies underlined the association between Neuro-developmental outcome and brain‐derived neurotrophic factor level in relation to feeding practice in early infancy. They assessed milk neurotrophins (nerve growth factor (NGF) and brain derived neurotrophic factor (BDNF) as a possible candidate for enhanced cognition and postnatal brain development in breastfed infants.

Several studies are investigating the biomarker concentrations of S100B protein, BDNF, andActivin A in human milk that seem to be the most trustable and promising developmental markers. Activin A and S100B protein have been recently found to be both trophic and brain damage markers, supporting the notion of the undisputed beneficial effects of breast milk feeding. Results open up a new cue on the use of these markers in perinatal medicine as a key protein for investigations focusing on fetal/neonatal development.

In addition Milk fat globule membrane (MFGM) components provide neurological benefits to the rapidly developing infant in terms of significantly improved performance in the Bayley cognitive, language and motor domains at 12 months of age and general improved cognitive outcomes. This supplementation of MFGM components in infant formula may narrow the gap in cognitive performance and infection rates between breastfed and formula-fed infants.

Human milk is complex and dynamic fluid, and several important methodological considerations are vital when exploring the association between HM composition and infant growth or neurodevelopmental outcomes. Considering also that the possible linkage between outcomes and HM components remains a pure speculation due to the absence of studies demonstrating the intestinal passage/absorption of these molecules, future studies longitudinal, well designed, with gold standard methodologies, are warranted. To obtain high quality and comparable results, the use of optimal and standardized sampling and quantification methods is essential. Human milk composition changes extensively according to stage of lactation and even within a single feed, highlighting the importance of standardized collection methods and longitudinal study designs. Few studies included in this review used identical human milk collection methods or collected human milk at similar stages of lactation, limiting comparability between studies or generalization of the results. For future research, trials that randomize lactating mothers to, for example, a multiple micronutrient or a fatty acid supplement and investigate how the supplement alters human milk composition along with the effect on infant growth and development is warranted.

In addition to methodological issues, careful considerations of several maternal and environmental determinants of human milk composition are needed in future studies, in fact it is currently unknown how maternal or environmental factors influence the concentration of these crucial biomarkers.

## Data Availability

Not applicable.

## Abbreviations

ADPH: Adipophilin
BDNF: Brain-derived neurotrophic factor
DHA: Docosahexaenoic acid
FABS: Fatty acid binding proteins
GDNF: Glial cell line-derived neurotrophic factor
HM: Human milk
HMOs: Human milk oligosaccharides
IGF-1: Insulin-like growth factor-1
LADH: Lactadherin
LCPUFA: Long-chain polyunsaturated fatty acids
MFGM: Milk Fat Globule Membrane
NCAM: Neural cell adhesion molecule
TAGs: Triacylglycerols
TGF-beta: Transforming growth factor beta
TNF: Tumor necrosis factor
VLBW: Very Low Birth Weight
XOR: Xanthine oxidoreductase
